# Gamasoidosis (bird mite dermatitis): *dermanyssus gallinae* in a young boy

**DOI:** 10.11604/pamj.2018.30.144.14651

**Published:** 2018-06-20

**Authors:** Marcos Davi Gomes de Sousa, Fred Bernardes Filho

**Affiliations:** 1 Instituto Nacional de Infectologia Evandro Chagas (INI-Fiocruz), Rio de Janeiro, Brazil; 2 Dermatology Division, Department of Medical Clinics, Ribeirão Preto Medical School, University of São Paulo, Ribeirão Preto, Brazil

**Keywords:** Mite infestations, pest control, dermatitis

## Image in medicine

A 6-year-old boy presented with a 4-day history of a pruritic eruption associated with excoriated erythematous papules over his limbs. He had no known medical conditions and was on summer school vacation 15 days ago. He had a healthy domestic dog without fleas or ticks. On physical examination, multiple erythematous papules and lesions with corkscrew morphology on his legs and feet were observed. In a visit by the first author to the site, many mites on the internal walls and windows, as well as the beds, were observed. Several nests of sparrows (*Passer domesticus*) were found and removed from the roof. Three representative mites were collected and subsequently were examined precisely; the causative agent of disturbance was recognized as *Dermanyssus gallinae*. The patient was treated with topical hydrocortisone 1% cream and loratadine (5 mg, once daily) for three days. Avian mites infest humans accidentally, causing a dermatitis characterized by widespread pruritic lesions. It has a worldwide distribution, and is more common in spring and summer. It is haematophagous and feeds at night, leaving its host during the day to hide in close proximity to the bird's nest. Removal of these birds from nesting or roosting sites in the vicinity of afflicted patients, with or without subsequent acaricide treatment of the area, is sufficient to arrest infestations. Cutaneous reactions are nonspecic and difficult to diagnose without a degree of clinical suspicion. Clinically, the condition presents as itching, which intensifies at night, with development of erythematous maculopapules or papulovesicles. When the clinical presentation is nonspecific and the arthropod cannot be found, the only hope for a correct diagnosis lies in a thorough environmental anamnesis.

**Figure 1:**
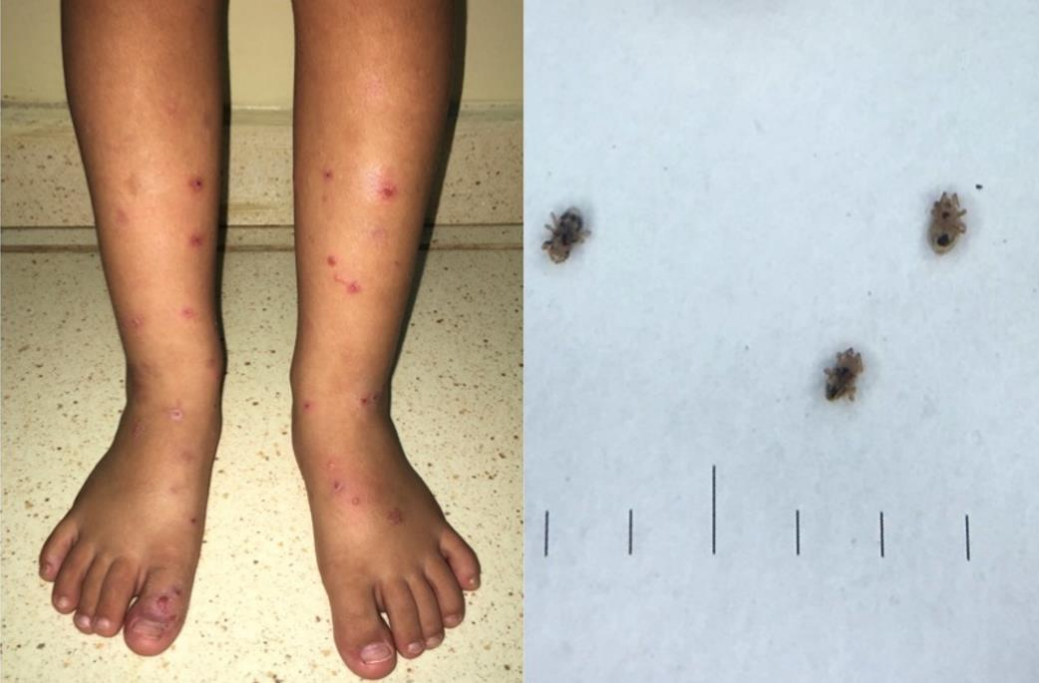
Multiple excoriated erythematous papules on the lower limbs of a 6-year-old boy (Dermanyssus gallinae dermatitis); dermoscopy with a handheld dermoscope (DermLite II Pro 3Gen (3Gen; SanJuan Capistrano, CA)) showing red mites found on the patient's bed

